# Widely Targeted Metabolomics Reveals the Effects of Soil on the Metabolites in *Dioscorea opposita* Thunb.

**DOI:** 10.3390/molecules28134925

**Published:** 2023-06-22

**Authors:** Lanping Yang, Yangyang Zhai, Zhenzhen Zhang, Zhenzhen Liu, Baohua Hou, Baobao Zhang, Zhenhui Wang

**Affiliations:** College of Medicine, Henan Polytechnic University, Jiaozuo 454000, China

**Keywords:** sandy soil, loessial soil, primary metabolites, secondary metabolites, differential metabolites

## Abstract

Chinese yam (*Dioscorea opposita* Thunb. cv. Tiegun), a type of homologous medicinal plant, mainly grows in sandy soil (SCY) and loessial soil (LCY). However, the effects of the soil on the metabolites in SCY and LCY remain unclear. Herein, this study aims to comprehensively elucidate the metabolites in SCY and LCY. A UPLC-MS/MS-based, widely targeted metabolomics approach was adapted to compare the chemical composition of SCY and LCY. A total of 988 metabolites were detected, including 443 primary metabolites, 510 secondary metabolites, and 35 other compounds. Notably, 177 differential metabolites (classified into 12 categories) were identified between SCY and LCY; among them, 85.9% (152 differential metabolites) were upregulated in LCY. LCY significantly increased the contents of primary metabolites such as 38 lipids and 6 nucleotides and derivatives, as well as some secondary metabolites such as 36 flavonoids, 28 phenolic acids, 13 alkaloids, and 6 tannins. The results indicate that loessial soil can improve the nutritional and medicinal value of *D. opposita*.

## 1. Introduction

*Dioscorea opposita* Thunb. cv. Tiegun, an important Chinese yam species, is one of the well-known edible and pharmaceutical foods in China [1]. This plant contains many active metabolites, such as polysaccharides, starch, lipids, flavonoids, polyphenols, amino acids, and organic acids [2,3,4]. These metabolites have various biological activities, such as antioxidant, antihypertensive, antidiabetic, impacting enzyme activities, regulation of spleen and stomach activity, enhancing immunity, antitumor, and antiaging [5,6,7]. *D. opposita* is used to treat chronic diarrhea, asthma, poor appetite, dry coughs, frequent or uncontrollable urination, diabetes, and emotional instability [2,8,9].

The term geoherb (also named as *Daodi* herb) refers to traditional Chinese medicine produced in specific regions (termed as *Daodi* districts) with a long history of clinical use with high quality and reliable clinical efficacy [10,11]. The geoherb production origins of *Dioscorea opposita* Thunb. cv. Tiegun have long been concentrated in Wen County (Jiaozuo City, Henan Province, China). Due to its geography, the south of Wen County is close to the Yellow River, and the soil is mainly sandy; the north of Wen County is near Taihang Mountain, and a small amount of silt in the Yellow River is mixed with the karst of Taihang Mountain, forming a unique loessial soil. Therefore, the *Dioscorea opposita* Thunb. cv. Tiegun planted in Wen County has better nutritional quality and medicinal value [12], as this plant grows on sandy soil (SCY) and loessial soil (LCY) [13]. The compound contents and nutrients of the plant vary due to the soil, environment, and climate, among other factors [2], and the soil exerts a strong influence on chemical composition [14]. Recent research has focused on the total polysaccharide, protein, monosaccharide, amino acid, and organic acid contents of SCY and LCY [2,12]. However, up to now, no comprehensive studies on the chemical composition or nutritional properties of SCY and LCY have been conducted.

Widely targeted metabolomics analysis [15], a novel method of metabolomics, combines the advantages of targeted metabolomics (high sensitivity) and nontargeted metabolomics (high throughput) [15,16], offering high throughput and ultra-sensitivity, as well as wide coverage of metabolites and accurate qualitative and quantitative analysis of thousands of metabolites in plant samples at once [16]. Currently, ultra-performance liquid chromatography–tandem mass spectrometry (UPLC-MS/MS)-based widely targeted metabolomic approaches have been successfully applied in medicine [17], agriculture [18], and food science [19]. Recent research has reported and characterized the metabolome profiling of eight Chinese yams by widely targeted metabolomics [8]. However, no information is available by using widely targeted metabolomics for revealing the effects of soil on the metabolites in *D. opposita*.

Therefore, this study aimed to comprehensively investigate the impacts of sandy soil and loessial soil on the composition and levels of primary and secondary metabolites of *D. opposita* using a UPLC-MS/MS-based widely targeted metabolomics approach. The results of this study may provide valuable information for the further development of the nutritional and medicinal value of *D. opposita*.

## 2. Results and Discussion

### 2.1. Widely Targeted Metabolomics Analysis of SCY and LCY

The metabolites of SCY and LCY were quantitatively analyzed using the multiple reaction monitoring (MRM) model (Supplementary Figure S1A). In order to ensure the reproducibility and reliability of the data, the superposition map of the total ion flow map (TIC) was detected using quality-control (QC) samples. The results in Supplementary Figure S1B showed a high rate of overlap, indicating that the reproducibility and reliability are good.

**Figure 1:**
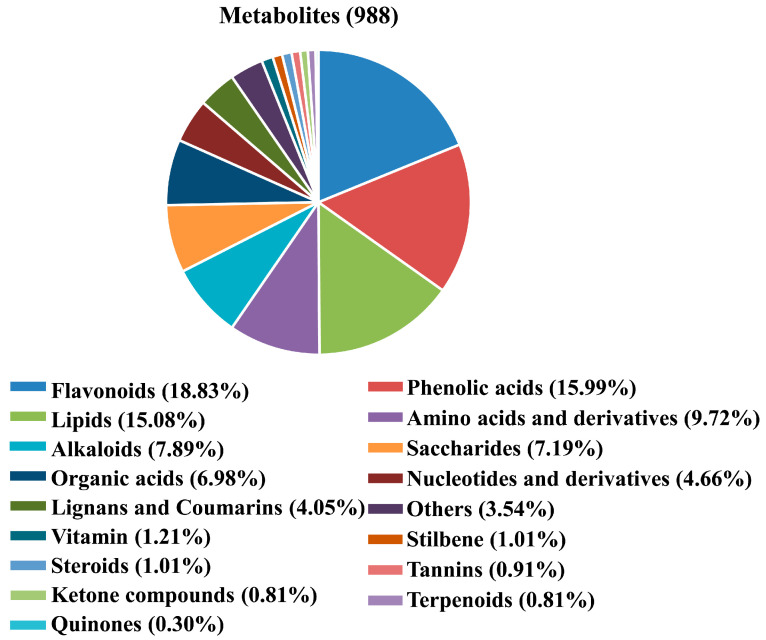
Pie chart of the metabolites identified from SCY and LCY.

A total of 988 metabolites were identified and annotated in SCY and LCY (Supplementary Table S1), which could be divided into 17 different categories, including 443 primary metabolites (149 lipids, 96 amino acids and derivatives, 71 saccharides, 69 organic acids, 46 nucleotides and derivatives, and 12 vitamins), 510 secondary metabolites (186 flavonoids, 158 phenolic acids, 78 alkaloids, 40 lignans and coumarins, 10 stilbenes, 10 steroids, 9 tannins, 8 ketone compounds, 8 terpenoids, and 3 quinones), and 35 other compounds. Based on the type and proportion of metabolites, flavonoids (18.83%), phenolic acids (15.99%), lipids (15.08%), amino acids and derivatives (9.72%), and alkaloids (7.89%) were the five dominant metabolites (Figure 1).

### 2.2. Multivariate Analysis of Metabolites in SCY and LCY

**Figure 2:**
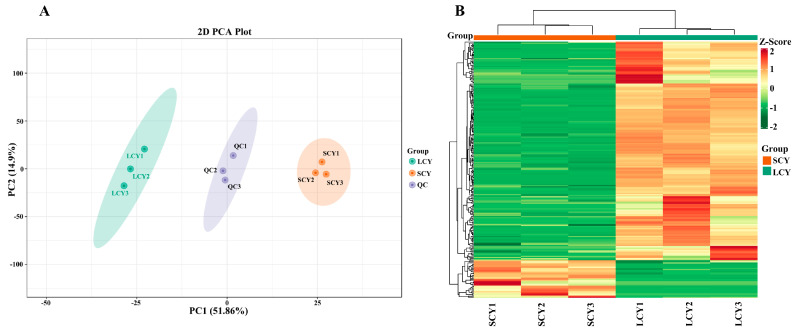
Multivariate analysis of identified metabolites. (**A**) Principal component analysis (PCA) analysis of metabolites identified from SCY and LCY. Equal volumes from SCY and LCY samples were mixed as quality control (QC) samples. (**B**) Hierarchical cluster analysis (HCA) of the metabolites identified from SCY and LCY.

In order to evaluate the overall differences between SCY and LCY samples, principal component analysis (PCA) and hierarchical clustering analysis (HCA) were performed. PCA could clearly separate the SCY and LCY samples from the QC samples (Figure 2A). Based on the first principal component (PC1, 51.86%) and the second principal component (PC2, 14.9%), the SCY and LCY samples were clearly divided into two categories, suggesting that each group had a distinct metabolite profile.

To eliminate the effect of quantity on pattern recognition, HCA was performed through a log10 transformation of peak areas for each metabolite. The SCY and LCY were clearly divided into two classes on the heatmap (Figure 2B), indicating significant differences in the content of metabolites between SCY and LCY; the results are consistent with those of the PCA.

**Figure 3:**
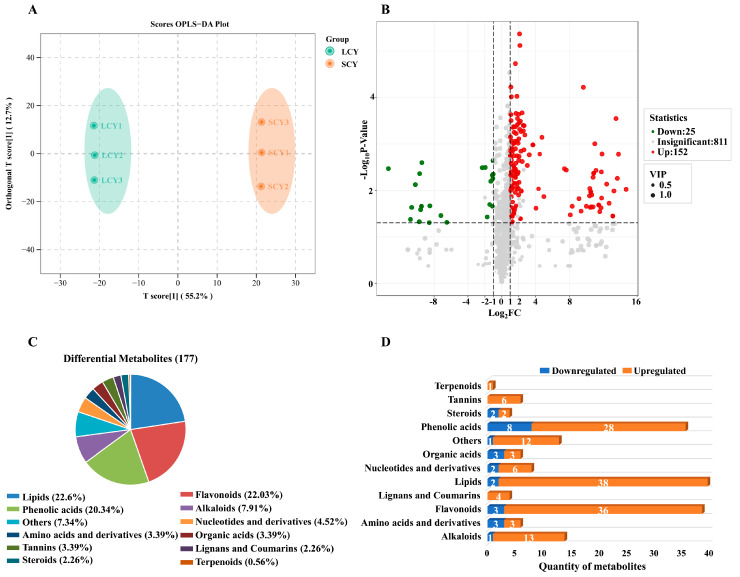
(**A**) Orthogonal partial least-squares discriminant analysis (OPLS-DA) model plot of the metabolites identified from SCY and LCY. (**B**) Volcano plot of the 177 differential metabolites identified. Differential metabolites were defined as metabolites with VIP ≥ 1, fold change ≥ 2 or ≤0.5, and *p*-value ≤ 0.05 in LCY relative to SCY. (**C**) Pie chart depicting the biochemical categories of differential metabolites identified between SCY and LCY. (**D**) Classification, change, and total number of differential metabolites between SCY and LCY.

The OPLS-DA model was used to compare the metabolic characteristics of SCY and LCY. The prediction parameters of the OPLS-DA model were R2X, R2Y, and Q2. The closer the three parameters were to 1, the more stable and reliable the model would be. In this model, the scores of R2Y and Q2 were 1 and 0.961 (*p* < 0.005), respectively, indicating that the model is appropriate (Supplementary Figure S2). SCY and LCY were separated in the OPLS-DA model (Figure 3A), demonstrating that there were differences in the metabolic profiles of SCY and LCY.

Based on the results of the OPLS-DA model, variable importance in projection (VIP) ≥ 1, fold change (FC) ≥ 2 or FC ≤ 0.5, and *p*-value ≤ 0.05, differential metabolites were selected in LCY relative to SCY. A total of 177 differential metabolites were identified between SCY and LCY (152 upregulated and 25 downregulated) (Figure 3B), which could be divided into 12 different categories, mainly including lipids (22.6%), flavonoids (22.03%), phenolic acids (20.34%), alkaloids (7.91%), and nucleotides and derivatives (4.52%) (Figure 3C). The changes in the metabolites of LCY were compared with those of SCY (Figure 3D). LCY significantly increased the contents of 50 primary metabolites, including 38 lipids, 6 nucleotides and derivatives, 3 amino acids and derivatives, and 3 organic acids, as well as 90 secondary metabolites, including 36 flavonoids, 28 phenolic acids, 13 alkaloids, 6 tannins, 4 lignans and coumarins, 2 steroids, and 1 terpenoid. Figure 3D clearly shows that lipids, flavonoids, phenolic acids, and alkaloids were four dominant upregulated differential metabolites in LCY relative to SCY. These results demonstrate that the metabolites between SCY and LCY were significantly different.

### 2.3. Differential Metabolite Analysis between SCY and LCY

**Figure 4:**
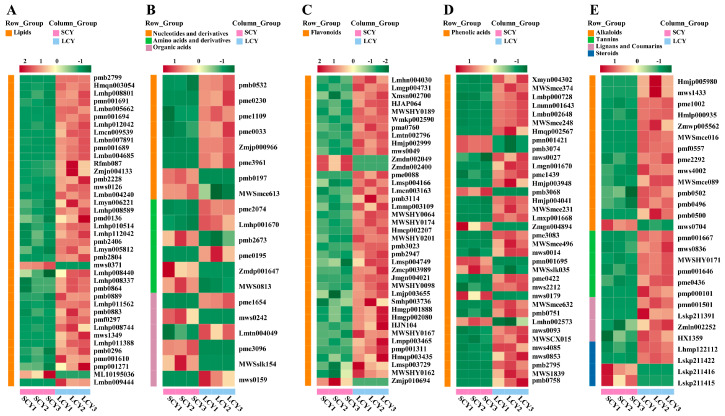
Thermograph of differential metabolites between SCY and LCY. (**A**) Lipids. (**B**) Nucleotides and derivatives, amino acids and derivatives, and organic acids. (**C**) Flavonoids. (**D**) Phenolic acids. (**E**) Alkaloids, tannins, lignans and coumarins, and steroids.

The differential metabolites between SCY and LCY were grouped using hierarchical clustering analysis. The heatmap revealed that LCY had a more significant impact on lipids (Figure 4A), nucleotides and derivatives, amino acids and derivatives, organic acids (Figure 4B), flavonoids (Figure 4C), phenolic acids (Figure 4D), alkaloids, tannins, lignans and coumarins, and steroids (Figure 4E) than SCY did.

#### 2.3.1. Differences in Primary Metabolites between SCY and LCY

##### Lipids

The lipids of free fatty acids, lysophosphatidylcholine (LysoPC), lysophosphatidylethanolamines (LysoPE), glycerol ester, and sphingolipids were examined. LCY led to significant increases in the types and contents of lipids (Figure 4A). A total of 40 differential metabolites of lipids were identified and annotated (Supplementary Table S2). Except for cis-4,7,10,13,16,19-docosahexaenoic acid and 3-dehydrosphinganine, the content of 38 lipids (including 19 free fatty acids, 8 glycerol esters, 7 LysoPEs, 4 LysoPCs, and 2 sphingolipids) were increased significantly (*p* < 0.05) in LCY. These results indicate that LCY significantly increased the contents of the primary metabolites of free fatty acids, glycerol ester, LysoPE, LysoPC, and sphingolipids than SCY did.

Especially, a total of 8 lipids, including 6 free fatty acids (12,13-epoxy-9-octadecenoic acid, E,E,Z-1,3,12-nonadecatriene-5,14-diol, 9-hydroxy-12-oxo-15(Z)-octadecenoic acid, 5S,8R-DiHODE, 1-eicosanol, and ricinoleic acid), 1 LysoPC (LysoPC 19:0), and 1 sphingolipid (dihydrosphingosine), were newly generated in LCY compared with those in SCY (Table 1). These metabolites were reported to have important functional and biochemical properties. For example, 12,13-epoxy-9-octadecenoic acid protects renal cells against decreases in mitochondrial and transport functions induced by hypoxia/reoxygenation [20]. E,E,Z-1,3,12-nonadecatriene-5,14-diol was identified for potential bioactive compounds as a SARS-CoV-2 inhibitor [21]. 1-Eicosanol exhibited antibacterial, antifungal, and antitumor activities [22,23]. It was reported that 1-eicosanol extracted from the acetate fraction of Leea indica could inhibit the growth of various cancer cell lines [23]. Ricinoleic acid exhibits analgesic, anti-inflammatory, and antinociceptive properties, as well as improvements in protein and energetic metabolism [24]. Dihydrosphingosine could promote keratinocyte differentiation and induce ceramide production, and it showed anti-inflammatory and antimicrobial activities [25].

##### Other Differential Primary Metabolites

A total of 20 other differential primary metabolites (including 8 nucleotides and derivatives, 6 amino acids and derivatives, and 6 organic acids) were identified (Figure 4B and Supplementary Table S2). It was reported that dietary sources of nucleotides have essential effects on immune function and maintain optimal physiological function [15]. The nucleotides and derivatives of inosine 5′-monophosphate, adenosine, guanine, hypoxanthine, vidarabine, and 2′-deoxyadenosine were upregulated in LCY (Table 1), with 2.11-, 4.75-, 2.45, 2.48-, 4.80-, and 1777.78-fold increments, respectively. Particularly, 2′-deoxyadenosine was newly generated in LCY vs. SCY. It was reported that 2ʹ-deoxyadenosine can inhibit ethanol-induced hepatocyte death in rats [26].

Amino acids and derivatives are not only important nutrients for the human body, but they also have various pharmacological activities, such as antioxidant [27], immune stimulation [28], and anti-inflammatory activities [29]. The amino acids and derivatives jasmonoyl-L-isoleucine, L-valyl-L-leucine, and L-cysteine were upregulated (with 26.92-, 2.96-, and 1422.22-fold increments, respectively); while 6-hydroxydopaquinone, γ-glutamyl-L-valine, and 5-oxoproline were downregulated (with 0.46-, 0.00-, and 0.48-fold decrements, respectively) in LCY relative to SCY. Jasmonoyl-L-isoleucine [30] and L-cysteine [31] are important active metabolites that regulate defense responses to various abiotic stress and developmental processes in plants. L-cysteine treatment is a safe and promising method to control postharvest brown rot, due to the activation of defense-related responses of the fruits to infection, and has a protective effect on postharvest fruits [31]. LCY resulted in a 26.9-fold increase in the content of jasmonoyl-L-isoleucine, and L-cysteine was newly generated in LCY.

The three organic acids jasmonic acid, abscisic acid, and phenylpyruvic acid were upregulated (with 8.13-, 2.16-, and 3077.78-fold increments, respectively), while suberic acid, aminomalonic acid, and triethyl citrate were downregulated (0.00-fold decrements) in LCY compared with SCY. Jasmonic acid is a simple phytohormone that regulates multifarious plant physiological processes, including development, growth, and defense responses to various abiotic and biotic stress factors [32]. Abscisic acid is a major phytohormone in plant abiotic stress resistance. In addition, a report has shown that abscisic acid treatment or nutrient-derived abscisic acid is beneficial in inflammatory diseases such as colitis and type 2 diabetes [33].

#### 2.3.2. Differences in Secondary Metabolites between SCY and LCY

##### Flavonoids

Compared with the metabolites in SCY, a total of 39 differential metabolites of flavonoids were identified in LCY (Figure 4C and Supplementary Table S2). Except for epicatechin-4′-O-β-D-glucopyranoside, epicatechin-3′-O-β-D-glucopyranoside, and retusin, the content of 36 differential flavonoids (including 14 flavonols, 9 flavanols, 7 flavones, 3 flavanonols, 2 flavanones, and 1 isoflavone) were upregulated. These results indicate that LCY significantly increased the contents of flavonoids than SCY did.

Flavonoids are important compounds in diet and disease treatment, with antioxidant and other biological activities [34]. Some of the highly accumulated flavonoids in LCY (such as luteolin, apigenin-7-O-glucoside, and catechin in Table 1) were shown to possess antioxidant bioactivities and other health benefits. For example, luteolin (1356.30-fold increment), a flavonoid found in many plants and herbs, has been identified to exhibit numerous biological activities, such as anti-inflammatory, antidiabetic, antioxidant, and neuroprotective ones [18]. Moreover, luteolin exerts anticancer properties on different cancers, such as colon cancer, lung cancer, prostate cancer, gastric cancer, glioblastoma, liver cancer, and breast cancer [35]. Apigenin-7-O-glucoside (2.13-fold increment) has diverse pharmacological activities, such as anti-inflammatory and neuroprotective properties [36]. Catechin (2.16-fold increment), epicatechin (3.08-fold increment), gallocatechin (4.59-fold increment), and epigallocatechin (2.51-fold increment) have anti-inflammatory, antioxidative, radical scavenging, chelating, and antiapoptotic properties [37,38].

##### Phenolic Acids

There are 36 differential metabolites of phenolic acids for SCY vs. LCY. Out of them, 28 differential phenolic acids were upregulated in LCY (Figure 4D and Supplementary Table S2), indicating that LCY is beneficial to the accumulation of phenolic acids.

Phenolic acids are bioactive phenolic compounds widely present in plants and foods, providing liver and cardiovascular protection and other biological functions, such as anti-inflammatory, antibacterial, anticancer, and antiviral activity [39]. Nine phenolic acids were detected in LCY but not in SCY, including hexahydrocurcumin, 4-hydroxycinnamic acid p-hydroxyphenethylamine, salicylic acid, dihydrodemethoxy curcumin, 2-methoxy-4-ethenylphenol, vanillin acetate, anisic acid-O-feruloyl glucoside, ethylparaben, and 4-methoxycinnamic acid (Table 1). LCY increased the contents of octahydrocurcumin, trans-5-O-(p-coumaroyl) shikimate, and sinapyl alcohol by 31.55, 22.5, and 16.51 folds, respectively (Supplementary Table S2). The upregulated phenolic acids in LCY have important roles in various biological functions. For example, hexahydrocurcumin and octahydrocurcumin possess several biological activities, such as antioxidant, anti-inflammation and antihypertensive [40], hepato-protective, and cardioprotective properties [41]. 4-Methoxycinnamic acid is a natural phenolic acid with multiple effects, such as neuroprotection and cancer inhibition [42]. It was reported that 4-methoxycinnamic acid would be a potential candidate for the treatment of schizophrenia, because of reducing schizophrenic-like behavior in mice and having fewer adverse reactions [43].

##### Other Differential Secondary Metabolites

A total of 28 other differential secondary metabolites (including 14 alkaloids, 6 tannins, 4 lignans and coumarins, and 4 steroids) for SCY vs. LCY were identified (Figure 4E and Supplementary Table S2). Compared with SCY, there were 13 out of 14 alkaloids, as well as all 6 tannins and 4 lignans and coumarins that were upregulated in LCY. Notably, five alkaloids (N-cis-feruloyltyramine, N-feruloyltyramine, cis-N-p-coumaroyltyramine, methyl nicotinate, and N-p-coumaroyl-N’-feruloylputrescine) and two lignans (fargesin and matairesinol) were newly detected in LCY but not in SCY (Table 1). The differential steroids △5-pregnene-3β,17α,20(S)-triol glucoside and sileneoside C were upregulated (with 2.20- and 3.98-fold increments, respectively), while nusilsterone and 26-hydroxyintegristerone A were downregulated (with 0.00- and 0.01-fold decrements, respectively). (Table 1) These results indicate that LCY could increase the contents of secondary metabolites of alkaloids, tannins, and lignans and coumarins relative to those in SCY.

These upregulated metabolites in LCY have important roles in various biological functions. For example, feruloyltyramine showed antioxidant and anti-inflammatory activities [44]. Cis-N-p-coumaroyltyramine, as a potential xanthine oxidase ligand, has good antioxidative and antihyperuricemic activities [45]. Fargesin exerts its anti-inflammatory effects and is commonly used in the treatment of allergic rhinitis, inflammation, sinusitis, and headache [46]. The anticancer activity of matairesinol has been reported in various types of cancers, including prostate, breast, cervical, and pancreatic cancer [47].

### 2.4. KEGG Annotation and Enrichment Analysis of Differential Metabolites

**Figure 5:**
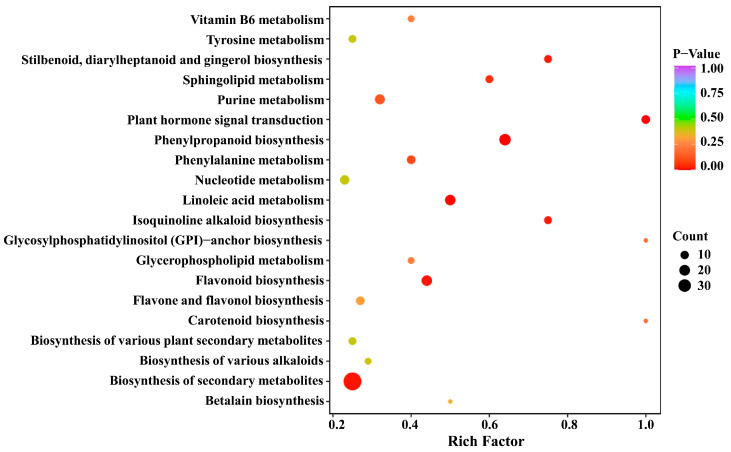
Kyoto Encyclopedia of Genes and Genomes (KEGG) pathway enrichment of the 177 differential metabolites.

The pathway enrichment analysis of 177 differential metabolites was carried out using the Kyoto Encyclopedia of Genes and Genomes (KEGG) database. As shown in Supplementary Figure S3, the differential metabolites were distributed in 49 metabolic pathways, and most differential metabolites were found in the metabolic pathways (70.18%) and the biosynthesis of secondary metabolites (52.63%). Subsequently, we conducted a KEGG pathway enrichment analysis (Figure 5) to identify the differences in metabolic pathways between SCY and LCY. The result revealed that biosynthesis of secondary metabolites, phenylpropanoid biosynthesis, flavonoid biosynthesis, linoleic acid metabolism, and plant hormone signal transduction were the most significant metabolic pathways with *p*-value ≤0.05.

## 3. Materials and Methods

### 3.1. Sample Preparation and Metabolite Extraction

Fresh *D. opposita* plants growing in sandy soil (SCY) and loessial soil (LCY) were obtained from Zhao Guozuo Village (34.941906 N, 113.047843 E), Wen County, Jiaozuo City, Henan Province, China. The growth conditions of SCY and LCY were completely the same (such as environment and climate), and only the soil texture was different. Three replicates of exclude-peel samples were collected. After being frozen in liquid nitrogen, all samples were stored at −80 °C. The widely targeted metabolomic analyses were carried out by Wuhan Metware Biotechnology Co., Ltd. (Wuhan, China) following previous reports with minor modifications [19,39]. Briefly, three technical replicates for SCY and LCY samples were freeze-dried in vacuum and then crushed using a mixer mill (MM 400, Retsch). The lyophilized powder (50 mg) was dissolved in 1.2 mL of methanol solution (70%), then mixed 6 times and vortexed for 30 s every 30 min. After centrifugation (12,000 rpm, 3 min), the extracts were filtrated (0.22 µm filter) before UPLC-MS/MS analysis. Quality-control (QC) samples were prepared by mixing 20 μL each of SCY and LCY extracts. A QC sample was performed for every three samples to ensure the repeatability of the measurement process.

### 3.2. UPLC and ESI-Q TRAP-MS/MS Conditions

The SCY and LCY sample extracts (4 μL for each) were analyzed using a UPLC-ESI-MS/MS system (UPLC, SHIMADZU Nexera X2, https://www.shimadzu.com.cn/, accessed on 10 May 2023; MS, Applied Biosystems 4500 Q TRAP, https://www.thermofisher.cn/cn/zh/home/brands/applied-biosystems.html, accessed on 10 May 2023) with an Agilent SB-C18 (1.8 µm, 2.1 mm × 100 mm) column. The mobile phase: solvent A (pure water with 0.1% formic acid), solvent B (acetonitrile with 0.1% formic acid). The gradient program was as follows: 5% B at 0 min, 95% B at 9 min and kept for 1 min, 5.0% B at 11.1 min and kept for 2.9 min. The flow velocity was 0.35 mL/min (column oven, 40 °C).

The effluent was alternatively connected to an ESI-triple quadrupole-linear ion trap (QTRAP)-MS with the following parameters: ion spray voltage (IS) 5500 V (positive ion mode)/−4500 V (negative ion mode); source temperature 550 °C; ion source gas I (GSI) 50 psi, gas II(GSII) 60 psi, and curtain gas (CUR) 25 psi; the collision-activated dissociation (CAD) was high. Instrument tuning and mass calibration were performed with polypropylene glycol solutions in the QQQ (10 μmol/L) and LIT (100 μmol/L) modes. The QQQ scans were acquired as MRM experiments with the collision gas (nitrogen) set to medium. The DP (declustering potential) and CE (collision energy) for individual MRM transitions were determined, with further DP and CE optimization. A specific set of MRM transitions were monitored for each period according to the metabolites eluted within this period.

### 3.3. Statistical Analysis

#### 3.3.1. Principal Component Analysis

Unsupervised principal component analysis (PCA) of the metabolites identified from SCY and LCY was performed using the statistics function prcomp within R (www.r-project.org, accessed on 10 May 2023). Before unsupervised PCA, the data were unit variance scaled (also known as Z-score normalization/auto-scaling). This method standardizes the data of SCY and LCY metabolites, according to the mean and standard deviation of the original data. The processed data accord with the standard normal distribution, that is, the mean value is 0 and the standard deviation is 1.

#### 3.3.2. Hierarchical Cluster Analysis

The hierarchical cluster analysis (HCA) results of the metabolites identified from SCY and LCY were presented as heatmaps with dendrograms, while the Pearson correlation coefficients (PCC) between the SCY and LCY samples were calculated using the cor function in R and presented as only heatmaps. Both HCA and PCC were carried out using the R package Complex-Heatmap. For HCA, the normalized signal intensities of the metabolites (unit variance scaling) in SCY and LCY were visualized as a color spectrum and displayed using the Euclidean distance metric.

#### 3.3.3. Differential Metabolites Selected

Supervised multiple-regression orthogonal partial least-squares discriminant analysis (OPLS-DA) was conducted to estimate the stability and reliability of the model. The VIP values were extracted from the OPLS-DA result, which also contained score plots and permutation plots and was generated using the R package MetaboAnalystR. The data were log transformed (log_2_) and mean centered before OPLS-DA. In order to avoid overfitting, a permutation test (200 permutations) was performed. Significantly difference metabolites between the SCY and LCY groups were determined using a variable importance in projection (VIP) value ≥ 1, fold change (FC) ≥ 2 (upregulated) or ≤0.5 (downregulated), and *p* < 0.05.

#### 3.3.4. KEGG Annotation and Enrichment Analysis

The differential metabolites between SCY and LCY were annotated using the Kyoto Encyclopedia of Genes and Genomes (KEGG) Compound database (http://www.kegg.jp/kegg/compound/, accessed on 10 May 2023), and then, the annotated metabolites were mapped to the KEGG Pathway database (http://www.kegg.jp/kegg/pathway.html, accessed on 10 May 2023) to obtain detailed pathway information. Pathways with significantly regulated metabolites were subjected to metabolite set enrichment analysis (MSEA). The *p*-values from the hypergeometric tests were used to assess their significance.

## 4. Conclusions

In this study, a UPLC-MS/MS-based widely targeted metabolomics approach was adapted to comprehensively identify and compare the primary and secondary metabolites of SCY and LCY. A total of 988 metabolites were detected, including 443 primary metabolites (149 lipids, 96 amino acids and derivatives, 71 saccharides, 69 organic acids, 46 nucleotides and derivatives, and 12 vitamins), 510 secondary metabolites (186 flavonoids, 158 phenolic acids, 78 alkaloids, 40 lignans and coumarins, 10 stilbenes, 10 steroids, 9 tannins, 8 ketone compounds, 8 terpenoids, and 3 quinones), and 35 other compounds. Notably, LCY significantly increased the contents of 50 primary metabolites, including 38 lipids, 6 nucleotides and derivatives, 3 amino acids and derivatives, and 3 organic acids, as well as 90 secondary metabolites, including 36 flavonoids, 28 phenolic acids, 13 alkaloids, 6 tannins, 4 lignans and coumarins, 2 steroids, and 1 terpenoid. The results indicate that loessial soil can improve the nutritional and medicinal value of *D. opposita*, which can provide valuable information for the further development of the nutritional and medicinal value of *D. opposita*.

## Figures and Tables

**Table 1:** Representative differential metabolites between SCY and LCY.

Compounds	VIP	*p*-Value	Fold_Change	Type
*Lipids*				
12,13-Epoxy-9-octadecenoic acid	1.35	0.00	804.07	Up
E,E,Z-1,3,12-nonadecatriene-5,14-diol	1.35	0.01	1592.59	Up
9-Hydroxy-12-oxo-15(*Z*)-octadecenoic acid	1.35	0.00	1859.26	Up
5S,8R-DiHODE	1.35	0.00	1859.26	Up
1-Eicosanol	1.35	0.00	3507.41	Up
Ricinoleic acid	1.35	0.01	3574.07	Up
LysoPC 19:0	1.35	0.02	1025.19	Up
Dihydrosphingosine	1.35	0.02	1770.37	Up
*Nucleotides and derivatives*				
Inosine 5′-monophosphate	1.33	0.00	2.11	Up
Adenosine	1.34	0.01	4.75	Up
Guanine	1.31	0.00	2.45	Up
Hypoxanthine	1.31	0.00	2.48	Up
Vidarabine	1.35	0.00	4.80	Up
2′-Deoxyadenosine	1.35	0.01	1777.78	Up
*Amino acids and derivatives*				
Jasmonoyl-L-isoleucine	1.32	0.00	26.92	Up
L-valyl-L-leucine	1.33	0.00	2.96	Up
L-cysteine	1.34	0.02	1422.22	Up
6-Hydroxydopaquinone	1.27	0.00	0.46	Down
γ-Glutamyl-L-valine	1.34	0.05	0.00	Down
5-Oxoproline	1.33	0.01	0.48	Down
*Organic acids*				
Jasmonic acid	1.34	0.00	8.13	Up
Abscisic acid	1.28	0.01	2.16	Up
Phenylpyruvic acid	1.35	0.03	3077.78	Up
Suberic acid	1.34	0.02	0.00	Down
Aminomalonic acid	1.35	0.00	0.00	Down
Triethyl citrate	1.34	0.03	0.00	Down
*Flavonoids*				
Luteolin (5,7,3′,4′-tetrahydroxyflavone)	1.35	0.01	1356.30	Up
Apigenin-7-O-glucoside (cosmosiin)	1.32	0.00	2.13	Up
Catechin	1.31	0.00	2.16	Up
Epicatechin	1.34	0.00	3.08	Up
Gallocatechin	1.30	0.00	4.59	Up
Epigallocatechin	1.33	0.00	2.51	Up
*Phenolic acids*				
Hexahydrocurcumin	1.35	0.01	13,000.00	Up
4-Hydroxycinnamic acid p-hydroxyphenethylamine	1.35	0.02	2829.63	Up
Salicylic acid	1.35	0.02	6622.22	Up
Dihydrodemethoxy curcumin	1.34	0.02	1511.11	Up
2-Methoxy-4-ethenylphenol	1.35	0.00	14,259.26	Up
Vanillin acetate	1.35	0.00	2262.96	Up
Anisic acid-O-feruloyl glucoside	1.34	0.03	666.67	Up
Ethylparaben	1.34	0.02	559.63	Up
4-Methoxycinnamic acid	1.35	0.00	170.74	Up
Octahydrocurcumin	1.33	0.01	31.55	Up
Trans-5-O-(p-coumaroyl) shikimate	1.11	0.00	22.50	Up
Sinapyl alcohol	1.32	0.02	16.51	Up
*Alkaloids*				
N-Cis-feruloyltyramine	1.35	0.04	8966.67	Up
N-feruloyltyramine	1.35	0.04	8966.67	Up
cis-N-p-coumaroyltyramine	1.35	0.02	2829.63	Up
Nicotinic acid methyl ester (methyl nicotinate)	1.35	0.00	2051.85	Up
N-p-coumaroyl-N’-feruloylputrescine	1.34	0.02	322.22	Up
*Lignans and coumarins*				
Fargesin	1.35	0.00	198.15	Up
Matairesinol (ISO2)	1.35	0.02	3392.59	Up
*Steroids*				
△5-Pregnene-3β,17α,20(S)-triol glucoside	1.33	0.00	2.20	Up
Sileneoside C	1.34	0.00	3.98	Up
Nusilsterone	1.34	0.02	0.00	Down
26-Hydroxyintegristerone A (ISO1)	1.34	0.03	0.01	Down

Note: All the differential metabolites are listed with variable importance in projection (VIP) ≥ 1, fold change (FC) ≥ 2 (upregulated) or FC ≤ 0.5 (downregulated), and *p*-value ≤ 0.05 (two-tailed Student’s *t* test) in LCY relative to SCY.

## Data Availability

Data are contained within the article and the Supplementary Materials.

## Supplementary Materials

The following supporting information can be downloaded at: https://www.mdpi.com/article/10.3390/molecules28134925/s1: Figure S1: Detection of metabolites with LC-MS/MS; Figure S2: OPLS-DA verification map; Figure S3: Kyoto Encyclopedia of Genes and Genomes (KEGG) pathway classification summary diagram; Table S1: Widely targeted metabolomics analysis of SCY and LCY; Table S2: Differential metabolites between SCY and LCY.
