# Supplementary material for: Widely Targeted Metabolomics Reveals the Effects of Soil on the Metabolites in Dioscorea opposita Thunb
Source: Molecules. 2023 Jun 22;28(13):4925. doi: 10.3390/molecules28134925 (PMC10343197; doi:10.3390/molecules28134925)
Supplement: Supplementary file 1 [file molecules-28-04925-s001.zip › Supplementary Figures.pdf]

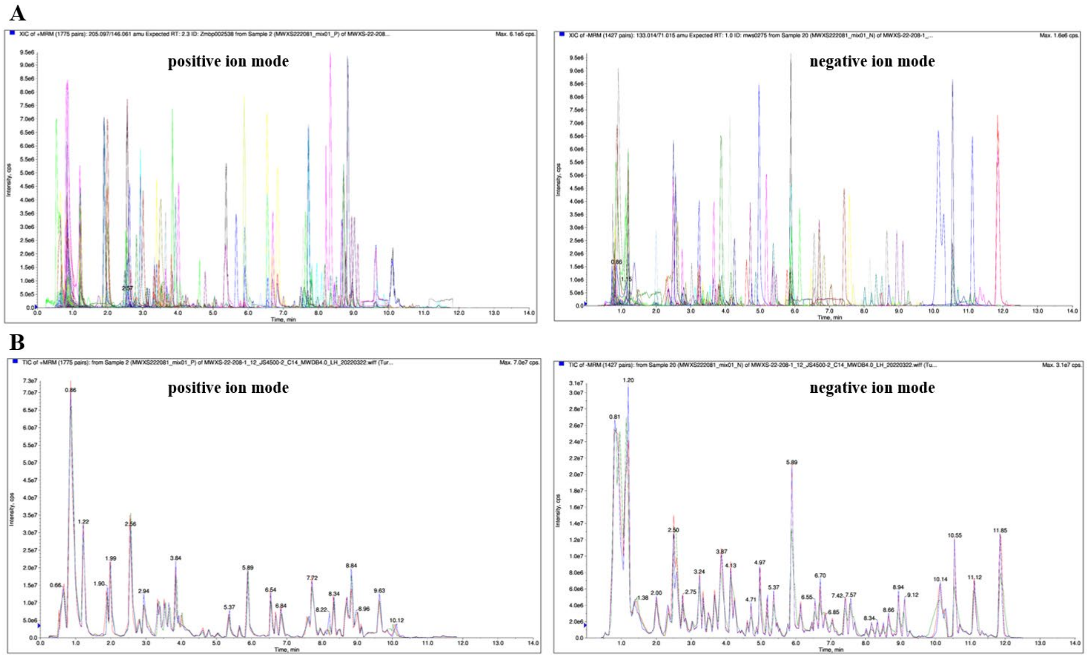

Figure S1. Detection of metabolites with LC-MS/MS.

A: Multipeak map of MRM metabolite detection (ion current spectrum of multi-material extraction, XIC)

B: Total ion current (TIC) overlapping map of quality control (QC) samples mass spectrometry results.

Note: The abscissa is the retention time of the metabolite detection (retention time, Rt). The ordinate is the ion current intensity of the ion detection (the intensity units are counts per second (cps)).

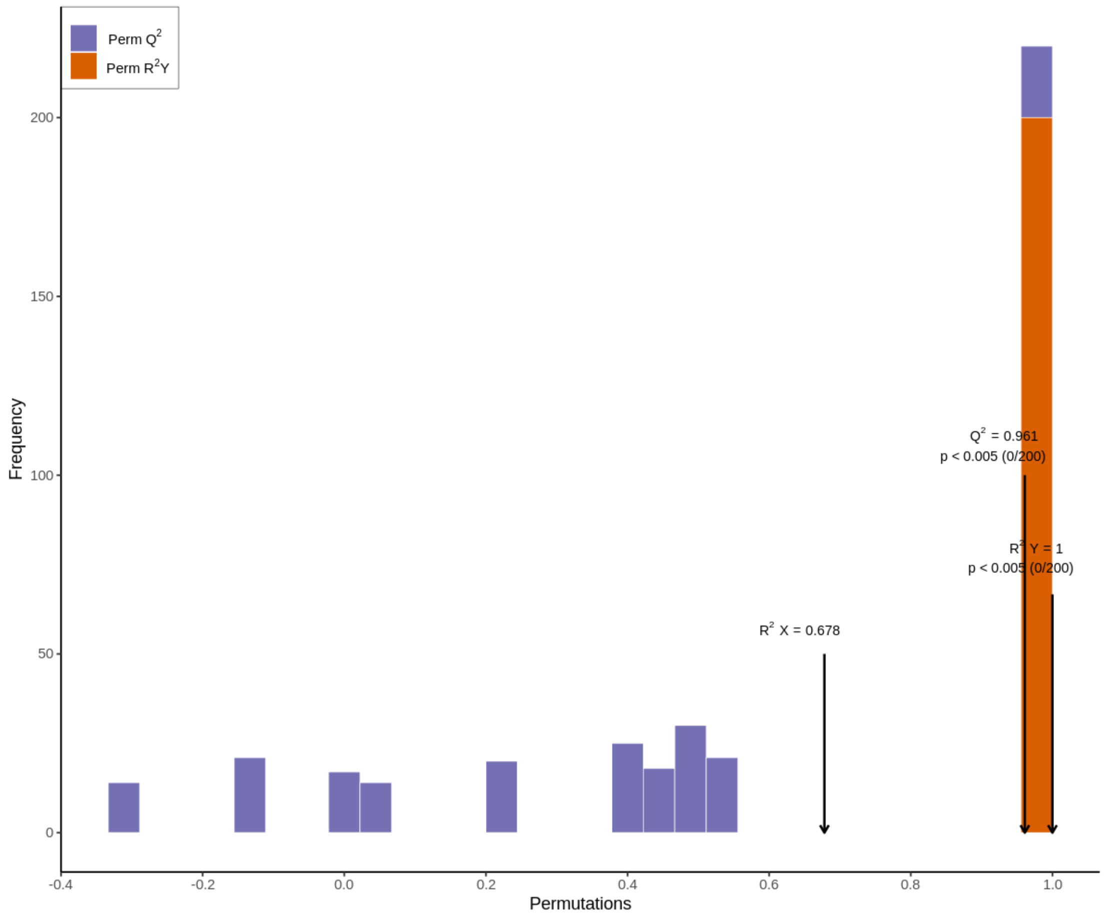

Figure S2. OPLS-DA verification map.

Note: Abscissa represents the values of model R^2Y and Q^2, and ordinate is the frequency of model classification effect in 200 random permutation and combination experiments. In the figure, the orange represents the random grouping model R^2Y, the purple represents the random grouping model Q^2, and the black arrow represents the R^2X, R^2Y and Q^2 values of the original model.

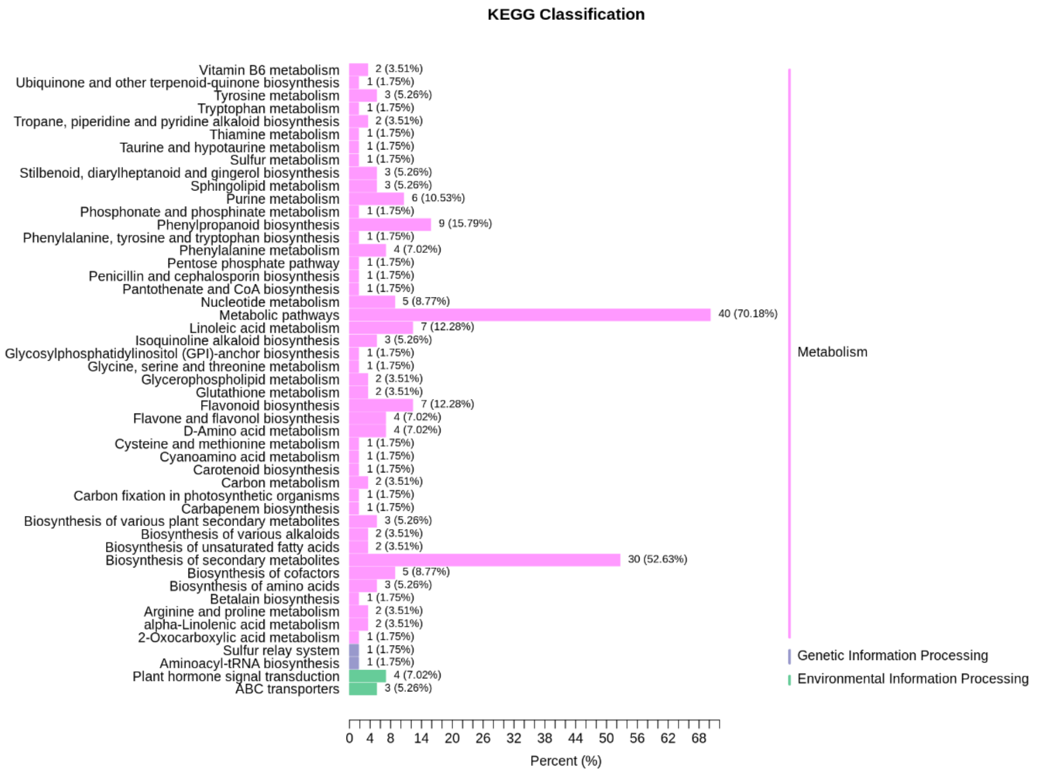

Figure S3. Kyoto Encyclopedia of Genes and Genomes (KEGG) pathway classification summary diagram.
